# Compton Camera and Prompt Gamma Ray Timing: Two Methods for *In Vivo* Range Assessment in Proton Therapy

**DOI:** 10.3389/fonc.2016.00080

**Published:** 2016-04-12

**Authors:** Fernando Hueso-González, Fine Fiedler, Christian Golnik, Thomas Kormoll, Guntram Pausch, Johannes Petzoldt, Katja E. Römer, Wolfgang Enghardt

**Affiliations:** ^1^Institute of Radiooncology, Helmholtz-Zentrum Dresden – Rossendorf, Dresden, Germany; ^2^OncoRay – National Center for Radiation Research in Oncology, Faculty of Medicine and University Hospital Carl Gustav Carus, Technische Universität Dresden, Dresden, Germany; ^3^Institute of Radiation Physics, Helmholtz-Zentrum Dresden – Rossendorf, Dresden, Germany; ^4^German Cancer Consortium (DKTK), German Cancer Research Center (DKFZ), Heidelberg, Germany

**Keywords:** proton therapy, range verification, *in vivo* dosimetry, Compton imaging, block detector, scintillation, prompt gamma ray timing

## Abstract

Proton beams are promising means for treating tumors. Such charged particles stop at a defined depth, where the ionization density is maximum. As the dose deposit beyond this distal edge is very low, proton therapy minimizes the damage to normal tissue compared to photon therapy. Nevertheless, inherent range uncertainties cast doubts on the irradiation of tumors close to organs at risk and lead to the application of conservative safety margins. This constrains significantly the potential benefits of protons over photons. In this context, several research groups are developing experimental tools for range verification based on the detection of prompt gammas, a nuclear by-product of the proton irradiation. At OncoRay and Helmholtz-Zentrum Dresden-Rossendorf, detector components have been characterized in realistic radiation environments as a step toward a clinical Compton camera. On the one hand, corresponding experimental methods and results obtained during the ENTERVISION training network are reviewed. On the other hand, a novel method based on timing spectroscopy has been proposed as an alternative to collimated imaging systems. The first tests of the timing method at a clinical proton accelerator are summarized, its applicability in a clinical environment for challenging the current safety margins is assessed, and the factors limiting its precision are discussed.

## 1 Introduction

In the first decades of the 20th century, during the rise of particle accelerators, physicists studied the interaction of fast charged particles with matter. The energy loss of *heavy* ions (as opposed to *light* electrons) within a target medium was described by Bethe’s stopping power formula (1). The ionization, namely, the Coulomb collisions where the accelerated ions strip out electrons of the atoms of the target, is the predominant loss mechanism for non-relativistic ion beams (2).

The engineering race toward high-energy accelerators endowed heavy charged particles a penetration depth in tissue comparable to the body dimensions. This opened up the possibility of using protons for medical applications, as neutrons, electrons, gamma, or X-rays had been applied before in the field of radiotherapy, which emerged after Röntgen’s X-ray discovery in 1895 (3).

In 1946, Wilson predicted the physical, in particular dosimetric, properties of a proton beam (4) for a therapeutic scenario and founded the field of proton therapy. The straight beam trajectory, the finite particle range, as well as the increase of the ionization density close to the stopping point, aroused the interest of the medical community. In the context of cancer treatment, this ionizing radiation was expected to damage the cells of the target tumor and eventually cause their death, while sparing most efficiently surrounding normal tissue.

The first experimental treatments were performed during the 1950s at Berkeley Radiation Laboratory, USA, and in Uppsala, Sweden (2, 5). However, it was not until 1990 that the first hospital-based proton facility in Loma Linda University (USA) was created. Since then, the number of therapy centers has increased steadily, and carbon or other ions have been also introduced. Nowadays, more than 15,000 patients are treated per year in around 50 facilities worldwide (5).

Several distinguishing features of accelerated protons are listed below:

- *The particle range*: the protons stop at a defined penetration depth, which depends on their initial velocity.
- *The Bragg peak*: the ionization cross section increases for low proton velocities, so that the dose deposition density is maximum close to the particle range (2). Conversely, the dose at the entrance point is minimum.
- *The distal penumbra*: beyond the particle range, the dose deposition falls steeply to zero.
- *The lateral penumbra*: the beam trajectory is straight and the spread in the transversal dimension due to multiple Coulomb scattering is small but increases steadily with depth [(6), p. 15].
- *Tissue composition*: the dose deposition curve and proton range are strongly dependent on the stopping power of the traversed tissue (on its density and composition).
- *Secondary products*: neutrons, annihilation photons, and prompt gammas are produced throughout the proton track. These secondary by-products release a small dose compared to the incident protons (2), and they can exit the patient. Neutron emission is focused in forward direction (7).

In theory, proton therapy has several advantages over conventional photon therapy:

- *The distal edge*: the steep dose gradient at the distal edge is promising for sparing critical organs close to the irradiated tumor. In contrast, the slowly falling depth dose curve of photons impedes that strategy.
- *The integral dose*: thanks to the Bragg peak, the dose can be focused on the tumor volume, and the damage on normal tissue before and beyond is minimized. For photons, the dose in healthy tissue can only be distributed on a larger volume by irradiating from several directions, but its integral is higher than for protons.

The main drawbacks compared to photons are:

- The capital expenditure on the facility construction and the higher clinical operating costs (8, 9).
- The lack of large clinical trials and evidence about the superiority of proton beam therapy for the majority of tumor entities (10). Whether or not the lower integral dose translates to better clinical outcome (11), e.g., less normal-tissue toxicity, is yet to be proven.
- Range uncertainties (12) of the proton beam due to intrinsic factors, such as patient or organ motion, as well as restricted knowledge about the tissue composition, are prone to yield severe differences with respect to the planned dose, specially at the distal edge, whereas photon plans are much less sensitive in this regard (13). To circumvent the risk, safety margins are applied and robust treatment plans are designed, at the price of an increase of the dose in the normal tissue compared to the dose-optimum (but more risky) plan.

These disadvantages question the cost-effectiveness of ion beam therapy and fuel the controversy about their clinical superiority [(14), chapters 2.11–2.13] over photon therapy. There is an urgent need for techniques that tackle one of the major weaknesses of proton therapy: the intrinsic range uncertainties, which limit the ultimate precision with which ion beams can be safely delivered. The most common sources of range errors are:

- Stopping power ambiguity due to degeneracy of Hounsfield values depending on tissue composition (15).
- Patient alignment errors.
- Anatomy changes between or during treatment fractions, as cavity filling, change of weight, tissue swelling, or tumor shrinkage.
- Organ motion in the thoracic and abdominal region.
- Biological factors (16).

**Figure 1:**
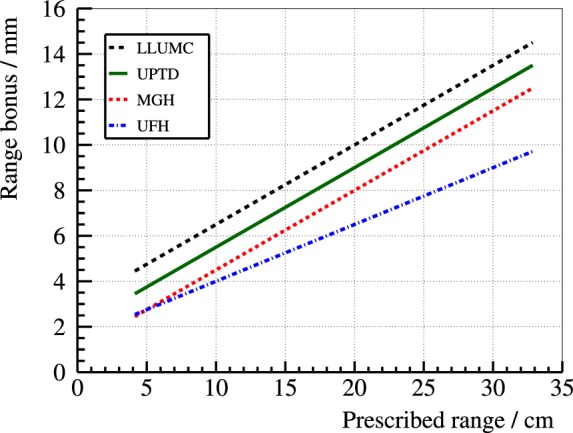
**Safety margins applied at different clinical proton therapy facilities: (3.5% + 3-mm) at Loma Linda University Medical Center (LLUMC) (3.5% + 2-mm) at Universitäts Protonen Therapie Dresden (UPTD) (3.5% + 1-mm) at Massachusetts General Hospital (MGH) and (2.5% + 1.5-mm) at University of Florida Health Proton Therapy Institute (UFH)**. *Range bonus* refers to the margin added to the prescribed range to ensure full tumor coverage even in the case of an undershoot. These centers may apply bigger margins in specific treatment scenarios (62).

The proton range is strongly dependent on the composition of the traversed tissue. Photons are less dependent on these factors, and the absence of a sharp edge constrains the maximum dose deviations due to target shifts or path composition variations. The absence of tools in clinical routine for measuring *in vivo* and in real time, the actual distal fall-off edge, together with the high sensitivity of the proton range to tissue composition, force medical physicists to add safety margins and apply field patching techniques in order to obtain a robust treatment plan (17). Notwithstanding the theoretically superior dose profile of ions, broad safety margins (Figure 1) waste substantially the outstanding traits of ion over photon beam therapy.

It should be emphasized that most cancers are treated successfully with surgery, electron or photon therapy, chemotherapy, brachytherapy, whereas proton therapy covers just a residual percentage (18). Still, the improvement in the accelerator technology, delivery systems, and the trend toward personalized medicine make proton beams an attractive alternative for certain patient ages and types of tumors. It is estimated that ~10% of cancer patients, especially children, would benefit from proton therapy (reduction of late side effects) compared to conventional techniques (18). Hence, proton therapy is still in the headlines, the number of facilities is increasing from year to year, and questions concerning the improvement of the technique and quality assurance are of great interest.

In this context, several groups across the world aim at an experimental device that measures the particle range and even the dose profile, preferably in real time (13). Numerous techniques have been proposed in the last two decades and are reviewed in Ref. (13, 19, 20). This paper fits into this context and summarizes two different methods for monitoring the dose delivery of proton beams in real time based on Prompt Gamma Imaging (PGI).

**Figure 2:**
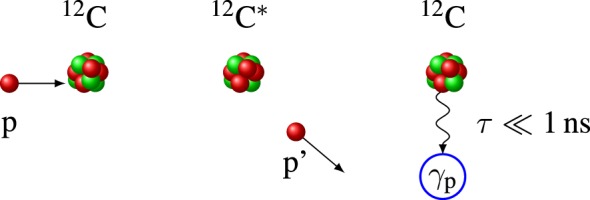
Schematic of a typical prompt gamma ray production process ^12^C(p,p′)^12^C*. Left: nuclear collision of a proton (p) with a ^12^C nucleus of the target. Center: the proton is scattered (p′) and the nucleus is left in an excited state ^12^C*. Right: the relaxation to the ground state ^12^C is accompanied by a 4.4-MeV prompt gamma emission *γ*_p_.

Prompt gamma rays, a by-product emitted in nuclear reactions along the proton track, cf. Figure 2, cover a broad energy spectrum with several prominent characteristic lines, cf. Ref. (21). The high gamma ray energy ensures that they can be detected outside the patient without severe attenuation. The spatial emission distribution correlates to the dose deposition map of the incident protons (21, 22) and provides an indirect measurement of the particle range. Such correlation is dependent on prompt gamma ray energy and tissue composition (23–26) and stems from the maximum of the nuclear cross section at low (~10-MeV) proton energies (27).

These gamma rays are *prompt*, i.e., they are emitted almost instantaneously after the collision, which is interesting for real-time range verification. The gamma ray production over 1-MeV is considerable, around 0.16 per proton (on average) at 160-MeV beam energy (28). The gamma ray emission rate depends on the beam current, duty cycle, and micro-time structure of the considered accelerator. Taking as an example the Cyclone^®^ 230 (C230) isochronous cyclotron of IBA (Louvain-la-Neuve, Belgium) and a realistic treatment plan with pencil beam scanning, the peak beam current is ≈2-nA, there are about 10^9^ gamma ray emissions per second, and 10^6^-s^−1^ events are registered in a ø2″ × 2″ LaBr_3_ scintillator at 30-cm distance (29). This large gamma ray rate, as well as the inherent neutron background, poses a serious challenge on the detector and electronics design. Note that the so-called neutron background is mostly indirect, due to the detection of gamma rays following neutron interactions or neutron captures in surrounding materials, rather than from the interactions of neutrons in the detector itself.

**Figure 3:**
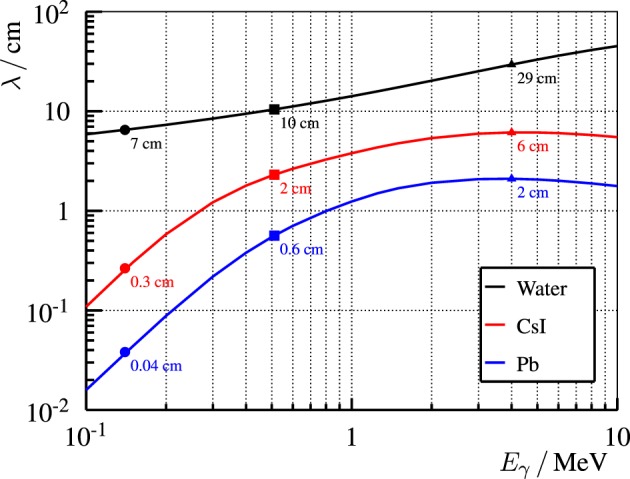
**Mean free path λ of photons of energy E_γ_ in different representative materials: water (body), CsI (detector), and Pb (collimator)**. λ is calculated as the inverse of the total attenuation factor *μ* that is extracted from (63). Explicit points at typical energies are drawn: 140-keV for ^99^Tc (nuclear medicine), 511-keV for PET, and 4-MeV for PGI.

In the field of nuclear medicine, commercial gamma cameras are used in clinical routine to obtain images of gamma ray distributions. Hence, one may think that the imaging of prompt gamma rays is not an issue, as the technology is already established. However, together with the detection rate and background, the high gamma ray energies and polychromatic energy spectrum prevent the direct use of the gamma camera as PGI device. In comparison, the gamma ray energies in nuclear medicine range between 80 and 511-keV. This significant difference is outlined in Figure 3: larger collimators and detectors are needed to absorb high-energy prompt gamma rays, normally after multiple interaction processes. For example, a 2-mm layer of lead has a 99% attenuation power for 140-keV photons, but only 9% for 4.4-MeV gammas; a 1-cm thick CsI crystal has a detection efficiency of 98% for the first and just 15% for the latter. Thick collimators reduce the system efficiency and deteriorate the image quality, whereas large detectors increase critically the system price and enlarge the footprint. Hence, alternative concepts are needed.

Dedicated PGI detector systems have been designed and tested in the last decade based on active or passive collimation. A pin-hole camera (30) is the pioneer approach to scan the prompt gamma emission distribution in a right angle to the beam track. Many research groups have performed experiments based on slit cameras at proton or carbon beams (31–37). The knife-edge-shaped camera has demonstrated the feasibility of millimeter range verification at clinical current intensities (38) in real time on a spot basis with realistic treatment plans and heterogeneous phantoms (39).

**Figure 4:**
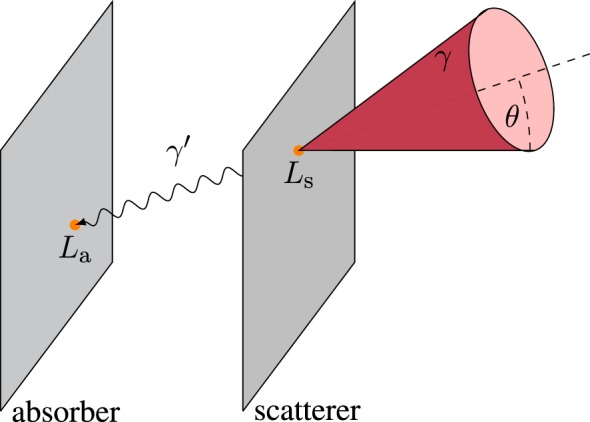
**Incoherent scattering event in a two-plane Compton camera**. The cone surface contains the possible incidence directions (any generatrix) of the initial photon (*γ*). It interacts with the scatterer plane and deposits an energy *L*_s_. The scattered photon (*γ*′) releases the rest of the energy *L*_a_ in the absorber. The line connecting both interaction points (in orange) is the propagation direction of *γ*′. This defines the axis (directrix) of the aforementioned cone, with half-opening (scattering) angle *θ* and vertex at the scatter point.

Among actively collimated systems, most efforts are concentrated on the Compton camera (40). It comprises multiple position sensitive gamma ray detectors, which are arranged in one scatterer and one absorber, or in several scatter planes. The prompt gamma rays reach the detectors, and the energy deposit as well as the point of interaction in each plane are measured, cf. Figure 4 for the two-plane camera. The Compton equation (41) relates the scattering angle *θ* to the initial (*E_γ_*) and final (*E_γ_*′) photon energies:

(1) $$\begin{array}{l} \;E_{\gamma }=L_{\text{s}}+L_{\text{a}}\\ \;E_{\gamma }\prime =L_{\text{a}}\\ \text{cos}\theta =1-{{\text{m}}_{\text{e}}\text{c}}^{2}\left(1/E_{\gamma }\prime -1/E_{\gamma }\right) \end{array}$$

where *L*_s_ and *L*_a_ are the energies released in scatterer and absorber, respectively, and m_e_c^2^ = 511-keV is the electron rest energy. In contrast to a slit camera, no collimation is needed in order to reconstruct the angle of incidence of the gamma ray, and two-dimensional (2D) or even three-dimensional (3D) images instead of one-dimensional (1D) profiles may be obtained. More single gamma rays and directions can be detected, but the condition of simultaneous interaction in the different camera stages limits the overall efficiency. Furthermore, the instrumentation requirements in terms of spatial, time, and energy resolution for the detectors of a Compton camera are especially high, and the reconstruction algorithm is complex and computationally intensive, as the incident direction cannot be recovered univocally for each event. Nowadays, a PGI Compton camera prototype demonstrating range verification in a clinical scenario is still a challenge several institutes aim at (42–47), and the only published experimental results at a proton beam are constrained to <2-MeV gammas (48, 49) or to beam currents far below the clinical case (50). Technical complexity, electronics expense, low coincident efficiency, high detector load, radiation background, and the elevated percentage of random coincidences are intrinsic hurdles that cast doubts on the applicability of this concept (19).

**Figure 5:**
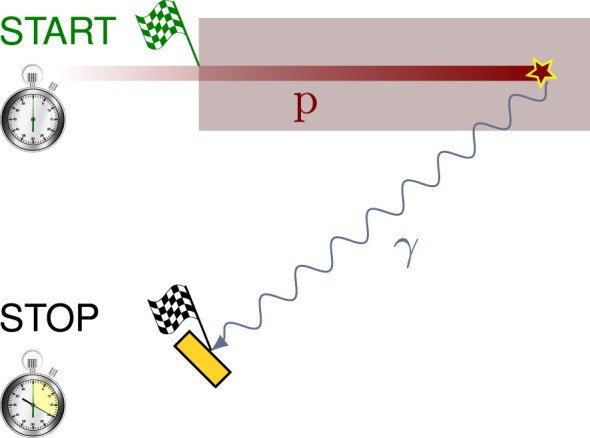
**Illustrative sketch of the PGT method**. The therapeutic proton p (the projectile) slows down as it penetrates the target and interacts with a nucleus, which emits a prompt gamma ray. The time between the entrance of the proton to the target (the start flag) and the arrival of the gamma ray to the detector (the stop flag at the yellow rectangle) encodes the proton transit time and gamma time of flight, which can be correlated to the depth of interaction of the proton (gamma emission point).

In the recent years, one can identify a trend toward less complicated PGI systems, at least concerning hardware. These may have a faster translation into clinical practice due to their lower price (35, 37, 51). The Prompt Gamma Ray Timing (PGT) method (28) is one of these novel approaches, which relies on a single monolithic detector with excellent timing resolution and no collimation. As a consequence of the measurable transit time of ions through matter, the detection times of prompt gamma rays encode essential information about their spatial emission point. Figure 5 illustrates this physical effect: the deeper the proton interaction (prompt gamma emission) point, the larger the proton transit time and time of flight of the gammas to the detector. Applying the Continuous Slowing Down Approximation (CSDA), the transit time can be derived mathematically (28) if the initial beam energy *E*_0_ and target composition are known. First, the proton energy variation per unit length yields

(2) $$\frac{\text{d}E}{\text{d}z}=-\rho (z)S(E)$$

where *E* is the kinetic energy, *ρ* (*z*) the mass density of the target at a depth *z*, and *S*(*E*) the stopping power, that depends on target material and thus indirectly on the depth *z*. The kinetic energy *E* of the proton at any depth *z* > *z*_0_ is

(3) $$E(z)=E_{0}-\int_{z_{0}}^{z}\;\rho (z\prime )S(E(z^{\prime }))\text{d}z^{\prime }$$

The relativistic velocity *v* of the particle is a function of the kinetic energy *E*:

(4) $$v(E)=c\;\sqrt{1-{\left(1+E/m_{\text{p}}{\text{c}}^{2}\right)}^{-2}}$$

where *m*_p_ is the proton rest mass. Finally, the equation of the proton transit time yields

(5) $$t_{\text{p}}(z)=\int_{z_{0}}^{z}\;\frac{1}{v(E)}\text{d}z^{\prime }=\int_{E(z)}^{E_{0}}\;\frac{1}{v(E)\;\rho (z^{\prime }(E))\;S(E)}\text{ d}E$$

where d*z*′ has been exchanged with d*E* using equation (2).

The low cost and small footprint of PGT makes this concept very tempting. A major limitation is that the time spectra are not only blurred by the resolution of the detector but also by the time width of the accelerated bunches. This implies that the PGT method is not applicable at all clinical accelerators: only to those with a specific micro-time structure. For the widespread accelerator C230 (5), the micro bunch time spread can reach up to 2-ns for clinical beam energies (29). Here, range shifts can be identified based on distribution momenta. It is under exploration whether PGT is only applicable for pencil beams or also for passively scattered ones. In order to know if other types of clinical accelerators are compatible with the PGT approach, the specific micro pulse structure has to be measured.

Rather than an in-depth review of the literature, this manuscript provides a summary of two separate topics developed within the collaborative framework of ENTERVISION (52): the characterization of detector components for the absorber plane of a clinical Compton camera and the first test of the PGT method with heterogeneous phantoms at a clinical proton center, corresponding to the publications (53) and (29), respectively.

## 2 Comparison of BGO and LSO Scintillation Detectors

Bi_4_Ge_3_O_12_ (BGO) and Lu_2_SiO_5_:Ce (LSO) scintillators are straightforward candidates to absorber detectors of a Compton camera aiming at PGI. These are used traditionally in Positron Emission Tomography (PET) scanners. Despite its higher price and 30% lower photoabsorption efficiency, LSO has gained importance due to its higher light yield and fast decay time. It is questionable if this conclusion can be transcribed to the PGI scenario. In order to assess the choice between the two options, benchmark experiments are conducted at different accelerators for comparing BGO and LSO detectors in terms of energy, spatial, and time resolution. Other factors, such as intrinsic radiation, absorption efficiency, and cost-effectiveness ratio, are also discussed.

### 2.1 Materials

**Figure 6:**
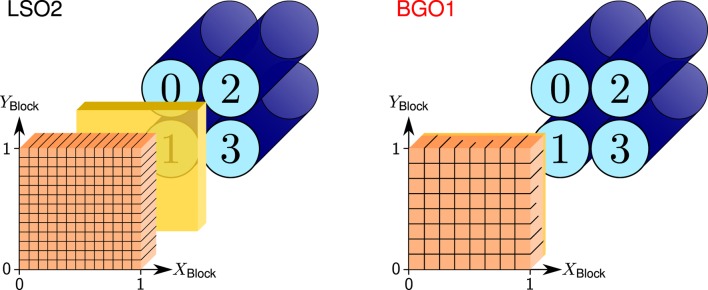
**Sketch of the LSO2 (left) and BGO1 (right) block detectors with the PMT numbering convention and crystal coordinate system, namely, the *X*_Block_ and *Y*_Block_ axis (relative position between 0 and 1)**. Crystals are depicted in orange, PMTs in blue, and the light guide in yellow. Reproduced with permission from Ref. (53).

The basic detection unit in commercial PET scanners is the block detector. It consists of a square matrix of segmented or pixellated scintillating crystals coupled to four light-sharing Photomultiplier Tubes (PMT), as depicted in Figure 6. The pixel where the gamma ray interacts can be calculated based on the ratio of light collected by each PMT. The block detectors used in this comparative study and their properties are listed in Table 1. They are named as LSO2 and BGO1 when referring to the concrete detector results, in contrast to LSO and BGO when speaking about general features of the scintillation materials.

**Table 1:** **Comparison of the properties of the different block detectors from Siemens Medical Solutions USA, Inc. Molecular Imaging Division, whose sketch is depicted in Figure 6**.

Detector name	LSO2	BGO1
Active volume (mm^3^)	52.7 × 52.7 × 20.0	52.7 × 52.7 × 20.0
Granularity	Independent pixels	Segmented crystal
Pixel matrix	13 × 13	8 × 8
Pixel dimensions (mm^3^)	4.0 × 4.0 × 20.0	6.4 × 6.4 × 20.0
Light guide	Coupled to block	Cut into block
Operating voltage	+900 V	+1350 V
Commercial scanner	Biograph PET/CT	ECAT EXACT 47 PET

*Spatial dimensions are given as height × width × depth. Reproduced with permission from Ref. (53)*.

### 2.2 Results

**Figure 7:**
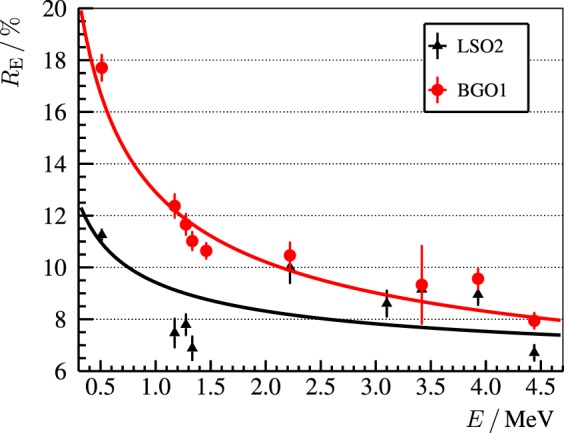
**Relative energy resolution *R*_E_ = Σ_E_/*E* of the LSO2 (black line) and BGO1 (red line) block detectors as a function of energy deposit *E***. Σ_E_ refers to the FWHM of the photopeak. The empirical fit to the experimental points is $R_{\text{E}}=(3.8\pm 0.3)\;\%/\sqrt{E/\text{MeV}}+(5.6\pm 0.3)\;\%$ for LSO2 and $R_{\text{E}}=(9.2\pm 0.5)\;\%/\sqrt{E/\text{MeV}}+(3.7\pm 0.4)\;\%$ for BGO1. Reproduced with permission from Ref. (53).

In Figure 7, the relative energy resolution *R*_E_ as Full Width at Half Maximum (FWHM) of the LSO2 and BGO1 detectors is compared. The energy resolution of LSO2 is better across the whole energy range. At 511-keV, *R*_E,LSO2_ ≈ 11% and *R*_E,BGO1_ ≈ 18%. Nonetheless, the differences are less pronounced for high-energy photopeaks (53, 54). At 4.4-MeV, *R*_E,LSO2_ ≈ 7% versus *R*_E,BGO1_ ≈ 8%. In other words, LSO2 excels at the PET scenario (below 1-MeV), whereas for the PGI scenario (above 2-MeV), the difference in performance is less significant, and the higher price of LSO does not imply a much better detector quality.

The reason for the comparable energy resolution at high energies is the following. The relative energy resolution *R*_E_ depends on two independent contributions: the statistical and the intrinsic one. The first one depends on the light yield and is proportional to the inverse square root of number of (collected) scintillation photons. The latter is due to non-linearity effects (55) and is dependent on the crystal structure. At low photon energies, i.e., the range of usual radioactive sources or in case of PET, the statistical contribution dominates over the intrinsic one. As LSO has a four times higher light yield than BGO, its energy resolution is significantly better. At high photon energies, i.e., the PGI energy range, the number of scintillation photons is larger, so that the statistical contribution is smaller and the intrinsic contribution starts to dominate. This intrinsic factor is comparable for BGO and LSO (54, 56, 57), which explains their similar performance concerning energy resolution at the PGI energy range.

**Figure 8:**
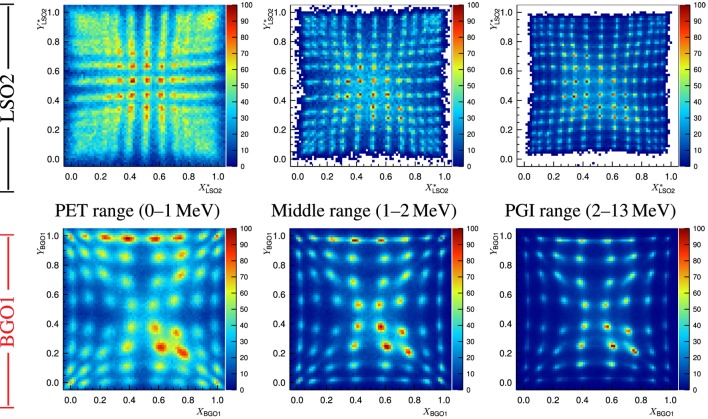
**Block detector flood map (top figure: LSO2 and bottom figure: BGO1) for different energy ranges at the ELBE accelerator (distortion correction applied for LSO2)**. Non-uniformities are due to the fact that the focused bremsstrahlung beam spot is smaller than the detector size as well as the different extension (number of bins) of each pixel spot in the map. Reproduced with permission from Ref. (53).

With respect to the spatial resolution, one can conclude that the discrimination power between pixels of the flood map increases with the energy range for both block detectors, cf. Figure 8. This effect is also due to the lower statistical relative uncertainty of events with high-energy deposit. The spots in the flood map of BGO1 are broader than those of LSO2 in any case, but become very sharp in the PGI range. This points out that one could segment the BGO1 block detector in, e.g., 13 × 13 instead of 8 × 8 and achieve the spatial resolution of LSO2 without jeopardizing the pixel discrimination in the flood map of the PGI range.

**Figure 9:**
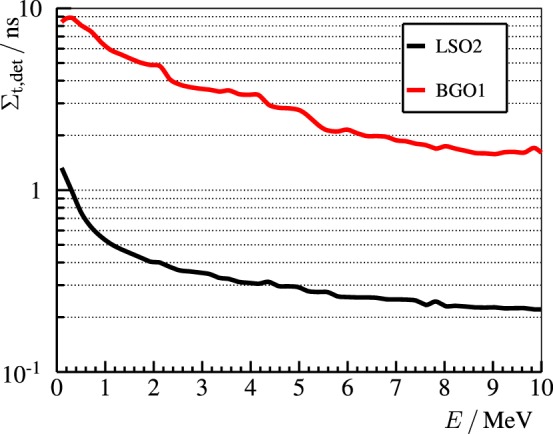
**Time resolution Σ_t,det_ (FWHM) of the LSO2 (black line) and BGO1 (red line) block detectors measured at the ELBE accelerator as a function of the released energy**. The experimental points are approximately reproduced by the curves $\Sigma_{\text{t},\text{det}}=(460\pm 10)\text{ ps}/\sqrt{E/\text{MeV}}+(80\pm 5)\text{ ps}$ for LSO2 and $\Sigma_{\text{t},\text{det}}=(4900\pm 500)\text{ ps}/\sqrt{E/\text{MeV}}+(10\pm 10)\text{ ps}$ for BGO1. Reproduced with permission from Ref. (53).

Regarding the time resolution, cf. Figure 9, the LSO2 detector beats BGO1 over the whole energy range thanks to the larger light yield and shorter decay time. A good time resolution is mandatory for a PGI Compton camera in order to suppress delayed radiation background (58). In order to analyze if the timing resolution of BGO1 is sufficient for this goal, we calculate the figure of merit *FoM*_BSR_ (59):

(6) $${\text{FoM}}_{\text{BSR}}=1-\frac{\sqrt{\Sigma_{\text{t},\text{det}}^{2}+\Sigma_{\text{t},\text{bunch}}^{2}}}{T_{\text{bunch}}}$$

where Σ_t,det_ is the detector time resolution as FWHM, Σ_t,bunch_ the bunch time spread (FWHM), and $T_{\text{bunch}}=f_{\text{bunch}}^{-1}$ the bunch period (the inverse of the radio frequency). This ratio measures the amount of background that can be suppressed thanks to timing measurements in a pulsed accelerator. In the case of the C230 machine, where *T*_bunch_ = 9.4-ns and Σ_t,bunch_ ≈ 2-ns (for 100-MeV protons) (29), the background suppression ratios for 4-MeV prompt gammas are *FoM*_BSR,LSO2_ = 84% and *FoM*_BSR,BGO1_ = 64%. LSO2 has undoubtedly better performance, but BGO1 could be also acceptable.

Other material features, such as the decay time and the intrinsic radioactivity, are worth to discuss. The decay time of BGO (7.5 times longer than LSO) implies a limit of around 300-kcps detector load. Taking into account the high rates expected in the PGI scenario, about 1-Mcps, one might be forced to reduce the area of the BGO block detectors or increase the distance to the beam axis. These rates are also quite challenging for the electronics and data processing. On the other hand, it is well known that LSO has a high intrinsic radioactivity below 1-MeV due to ^176^Lu, namely, through *β*^−^ decay and subsequent gamma ray cascade. The simultaneous detection of the electron (stopped in the LSO crystal) and the gamma ray (in the scatterer plane) could produce a significant fraction of false coincidences in a Compton camera.

## 3 Prompt Gamma Ray Timing with Heterogeneous Targets

The PGT method is a promising and novel method for range verification proposed by Golnik et al. (28) based on experiments with homogeneous phantoms at a research accelerator. To further explore its potential and the limitations that may appear when translating the concept to a realistic irradiation scenario, specific experiments at a clinical proton center with heterogeneous targets are conducted. The concrete goals are to test the robustness of the PGT method, its precision, and limitations, as well as the capability of detecting range shifts due to heterogeneities. Furthermore, the next steps toward a clinical PGT prototype are identified.

### 3.1 Materials

**Figure 10:**
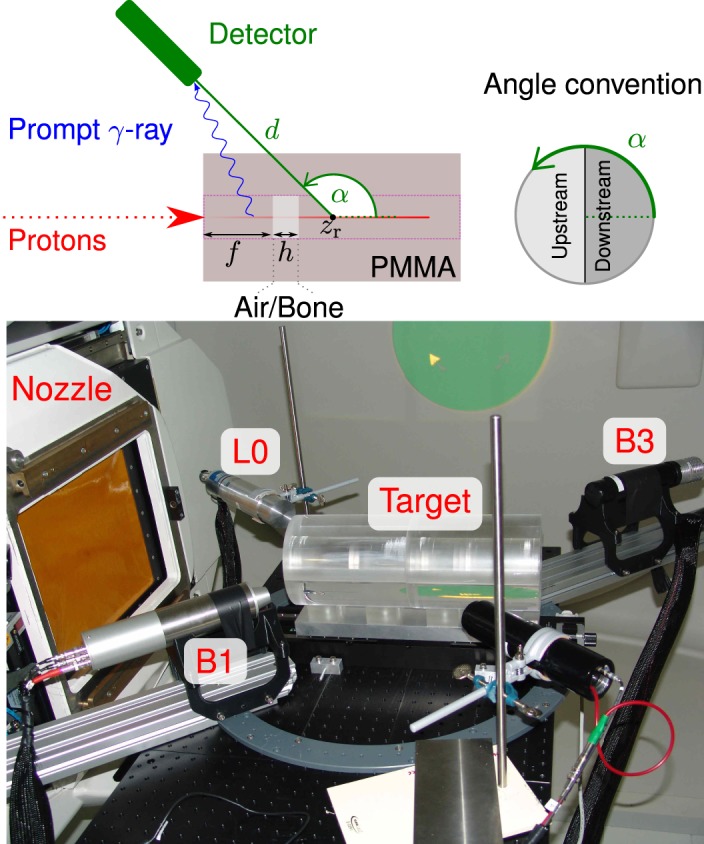
**Top: sketch of the experimental setup at the WPE proton therapy facility and of the longitudinal cross section of the target**. The accelerated protons bunched with 106 MHz collide with the PMMA cylindrical target. It contains a cavity or bone insert of thickness *h* located at a distance *f* from the beam entrance point. Bottom: photograph of the experimental setup with three detectors at different ring angles *α*. The linear stage on the center of the ring holds the two hollow joined half cylinders, in which PMMA, cavity, or bone slices can be inserted. The beam incidence is horizontal from the left, where the snout of the nozzle is seen. Reproduced with permission from Ref. (29).

The experiment is carried out at the Westdeutsches Protonentherapiezentrum Essen (WPE), Germany. This clinical proton center comprises a C230 cyclotron, with a radio frequency close to 106-MHz. A horizontal pencil beam (no scanning) in the gantry treatment room irradiates a cylindrical target, see Figure 10 (bottom). The inner shell contains slices of custom thickness and composition, so that different heterogeneous targets can be configured. Available materials are polymethyl methacrylate (PMMA), air (hollow slice), and cortical bone. The detectors listed in Table 2 are set up at an angle *α* and distance *d*, as described in Figure 10 (top). Whenever a target is thick enough to completely stop the impinging protons, we label it as *full* (for a given proton energy).

**Table 2:** **Monolithic scintillation detectors available in the experiment**.

Alias	Material	ø × Length	Rationale
B1	BaF_2_	[25:38] mm × 30 mm	Time resolution
B3	BaF_2_	48 mm × 31 mm	Time resolution
L0	LaBr_3_:Ce	2″ × 2″	Energy resolution

*B3 and L0 are cylindrical, whereas B1 is a tapered cone (for optimum time resolution). Reproduced with permission from Ref. (29)*.

### 3.2 Results

**Figure 11:**
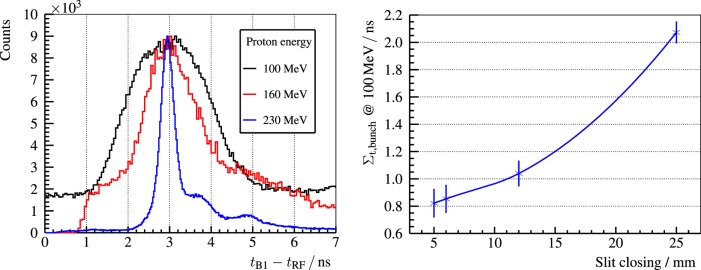
**Left: PGT spectra of the B1 detector with a thin PMMA target for three different proton energies and the usual slit closing (25-mm) at the WPE gantry**. Right: bunch time spread Σ_t,bunch_ at 100-MeV proton energy and a full PMMA target for the B3 detector. Reproduced with permission from Ref. (29).

The bunch time spread is an important limiting factor for the PGT method. In Figure 11 (left), the bunch width is characterized as a function of the proton energy, ranging from 350-ps at 230-MeV to 2-ns at 100-MeV. The time spread can be reduced up to a factor of two by adjusting the momentum spread limiting slits, cf. Figure 11 (right), the main component of the energy selection system of the C230 cyclotron (60).

**Figure 12:**
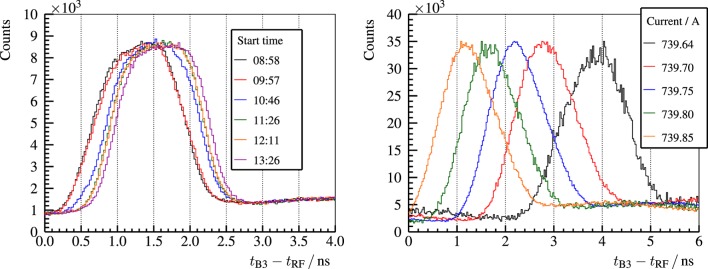
**Left: PGT spectra of the B3 detector, a homogeneous PMMA target and a proton energy of 230-MeV**. Independent redundant measurements of about 5-min with a separation of around 1-h are overlaid. Right: PGT spectra of the B3 detector, a full homogeneous PMMA target, and a proton energy of 160-MeV for different values of the main coil current of the cyclotron (detailed in the legend). Reproduced with permission from Ref. (29).

For the acquisition of PGT spectra, the detection time of the gamma rays with respect to the arrival of the protons to the target has to be measured. As usual in research accelerators, the radio frequency can be used as reference time for the bunch arrival (except for an offset). However, Figure 12 shows that this offset is not constant on a large time scale (29). These phase drifts of the proton bunch with respect to the RF signal may be caused due to temperature changes or main coil current instabilities, among other factors.

**Figure 13:**
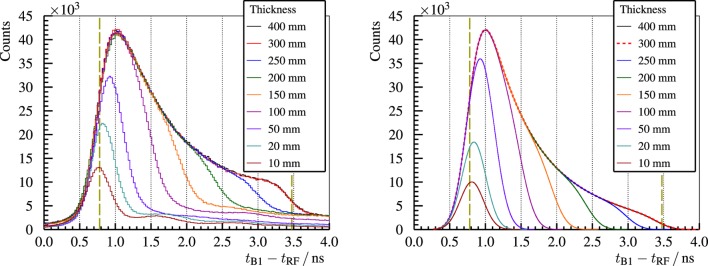
**Left: experimental PGT spectra of the B1 detector for 230-MeV protons and different PMMA target thicknesses**. Right: spectra calculated with the analytical simBox model (28). Both: the two vertical dashed lines refer to the expected front face and proton range positions. Reproduced with permission from Ref. (29).

With regard to homogeneous targets, the stacked target experiment is accomplished as follows: homogeneous PMMA targets of various thicknesses are irradiated with 230-MeV protons. The increase of the target thickness correlates to an increase of the area of the PGT distribution and a shift to the right in its mean value, due to the enlarged region of prompt gamma emission, as observed in Figure 13 (left). As the bunch time spread is significantly lower than the proton transit time, one can resolve the prompt gamma emission density as a function of the (timewise) depth with much less blurring than for 160 or 100-MeV proton energies. In Figure 13 (right), we calculate the PGT distributions according to the analytical simple Box (simBox) model (28). The shape is qualitatively similar to the experimental spectra, but the model is too simple to reproduce, e.g., the fall-off edge corresponding to the Bragg peak or the background radiation.

**Figure 14:**
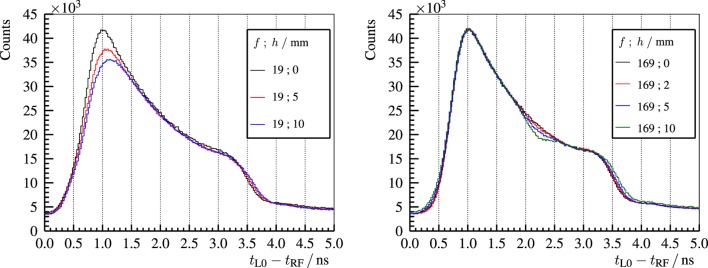
**Experimental PGT spectra of the L0 detector for 230-MeV protons and different air cavities inside the full (400-mm) PMMA target at *f* and *h* (front face position and thickness as described in Figure 10)**. Reproduced with permission from Ref. (29).

Concerning heterogeneous targets, air cavities of different thicknesses are placed at different depths inside a full PMMA target. The experimental results are shown in Figure 14. The deficit in the gamma ray production inside the air cavity can be identified as a dip in the PGT spectra at a time position and with a magnitude correlated to its location and thickness, respectively. The falling edge of the spectrum shifts steadily to the right according to the cavity thickness (beam overshoot).

**Figure 15:**
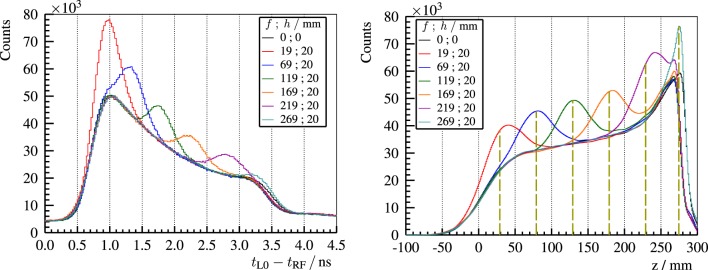
**Left: experimental PGT spectra of the L0 detector for 230-MeV protons and a bone insert inside the full (400-mm) PMMA target at *f* and *h* (front face position and thickness as described in Figure 10)**. Right: time to distance axis conversion from the left PGT spectra after application of stopping power, sensitivity, and gamma time-of-flight corrections as well as background subtraction. *z* refers to the depth with respect to the target’s front face (beam entrance point). Vertical dashed lines mark the centroid of the bump according to the simBox model. Reproduced with permission from Ref. (29).

An analogous experiment with a bone insert (20-mm thick) at different positions is carried out (29). The resulting PGT spectra are depicted in Figure 15 (left). An increase of the gamma ray production due to the higher density of bone is visible in the PGT spectrum at a time correlated to the insert position. A shift to the left in the falling edge of the distribution can be identified (undershoot) with respect to the homogeneous case. In Figure 15 (right), the PGT spectra are converted to depth profiles by using the transit time equation, cf. equation (5), and applying detector solid angle and gamma time-of-flight corrections.

**Figure 16:**
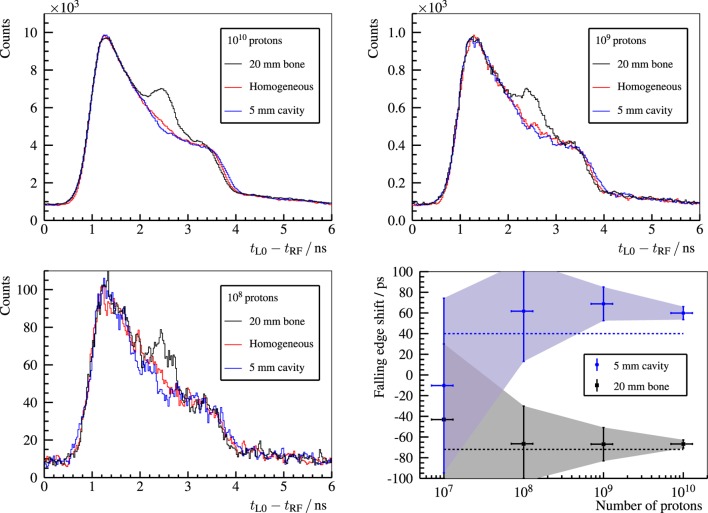
**Top row and bottom left plot: PGT spectra of the L0 detector for 230-MeV protons**. The homogeneous case (red curve) corresponds to a full PMMA target (400-mm). A heterogeneous slice is placed inside the full PMMA target at *f* = 169-mm and *h* = 5-mm (air cavity – blue curve) or *f* = 169-mm and *h* = 20-mm (bone insert – black curve), where *f* and *h* are the front face position and thickness, as described in Figure 10. The legend header contains the number of protons associated with each spectrum. Bottom right: shift of the falling edge with respect to the homogeneous case depending on the number of protons. The dashed lines depict the expected shift of the trailing edge according to the simBox model. Reproduced with permission from Ref. (29).

In Figure 16, for 230-MeV protons, the effect of a beam overshoot (air cavity) and undershoot (bone insert) on PGT spectra is compared with respect to a reference measurement (homogeneous PMMA target). Moreover, the detectability of the range shift based on the location of the trailing edge is analyzed as a function of the number of irradiated protons. For clinical treatment plans, the strongest spot in pencil beam scanning, which is usually at the distal edge, yields close to 10^8^ protons. This hints that a single detector is able to recover 5-mm range errors of the distal spot based on the PGT method with realistic beam currents at the C230 accelerator.

## 4 Discussion

Prompt gamma rays, produced in nuclear reactions of accelerated protons with tissue, are valid signatures for retrieving the range of therapeutic protons. Several imaging systems are under development in the scientific community. Among others, the Compton camera and the Prompt Gamma Ray Timing (PGT) method have been studied intensively during the last years in collaboration with the ENTERVISION project (52).

A Compton camera requires position-sensitive detectors with high resolution and efficiency. The characterization of different candidate detectors in the PGI energy range is mandatory for assessing the material choice based on measurements that complement previous simulations and textbook knowledge. BGO and LSO block detectors from commercial PET scanners are compared in terms of energy, spatial, and time resolution, as well as price, absorption efficiency, and intrinsic background. As expected, the overall performance of LSO is better, but BGO closes the gap in the PGI range. The reason is that the high gamma ray energies (compared to the PET scenario) and thus number of scintillation photons balances the lower light yield. In addition, BGO has a higher photoabsorption efficiency, no intrinsic radioactivity, and low cost. Hence, BGO is a competitive alternative for the absorber of a Compton camera, thanks to the superior cost-effectiveness ratio in the PGI field (53).

PGT is an innovative method for range assessment based on a low footprint detector setup at minimum expense. First tests at a clinical accelerator and with heterogeneous phantoms reveal the capability of measuring 5-mm range shifts (due to heterogeneities) for beam spots with 10^8^ protons (29). The bunch time spread is a crucial factor that affects the resolution of the PGT method. It depends on the delivered proton energy and the settings of the energy selection system. Furthermore, bunch phase drifts are found throughout the experiment, which pose a challenge on the robustness of the PGT method on a large time scale. Hence, it is advisable to introduce a proton bunch monitor (60, 61) that measures the bunch time structure as well as the potential phase drifts. A larger detector load and acquisition throughput are mandatory to improve the number of gamma rays detected per proton, so that statistically significant conclusions on range errors can be drawn for more spots of the treatment plan. Quantification of the range shifts based on more sophisticated models or simulations are also necessary. Experiments with upgraded detectors and electronics, realistic treatment plans, and anthropomorphic phantoms are ongoing. These are the next steps toward clinical translation and development of a first robust prototype.

## Author Contributions

All authors were involved in the conceptual design, management, support, and accomplishment of the experiments reviewed in this paper as well as in the critical revision of the data analysis and obtained results.

## Conflict of Interest Statement

The authors declare that the research was conducted in the absence of any commercial or financial relationships that could be construed as a potential conflict of interest.
